# Restrictions in the Use of Unproved Remedies

**Published:** 1895-11-30

**Authors:** 


### The Hospital

An Institutional Journal of

The Medical Sciences and Hospital Administration, with

AN EXTRA NURSING SUPPLEMENT.

Vol. XIX.—No. 479.] [Nov. 30, 1895.

### Restrictions in the Use of Unproved Remedies.

Early in October the Italian Minister of the Interior published a decree forbidding the general sale and use of Professor E. Maragliano's anti-tuberculous serum until it should, after sufficient evidence of its value and safety had been adduced, be approved by the Superior Council of Health. No restriction was, however, placed upon its free use for experimental purposes by Professor Maragliano himself. Since that date the Director of Public Health has approved the temporary interdict of the Minister of the Interior, and so, for the time being, the use of the serum for any purpose connected with human life is forbidden, except under Professor Maragliano's own direct responsibility. Needless to say this interdict has given satisfaction to some and offence to others both within and without the medical profession in Italy.

With regard to the serum itself, as prepared by Maragliano, there is little at present to be said. It is an anti-tuberculous serum, as Koch's was an anti-tuberculous fluid, and up to the present, like Koch, Maragliano has not disclosed either its exact composition or the method of its preparation. It is true that he has made public certain facts regarding its constituent materials, and it was expected that full disclosures and a complete exposition would be forthcoming at the Italian Congress of Internal Medicine held a few weeks ago. No such complete exposition was, however, made, and much dissatisfaction on the part of the members was freely expressed.

The object of reproducing these facts here is to consider the whole question of the introduction of new and unproved remedies to general use. In this question it is obvious that not the medical profession alone but the general public is deeply interested. There may be here and there an enthusiastic experimenter who holds that it is the privilege of the lay world to supply subjects for experimentation. Manifestly, however, such views cannot be entertained by responsible medical practitioners, and they certainly are not entertained by the conductors of this journal. Nevertheless, it has to be recognised that all medical practice is, to a certain extent and in a certain sense, a matter of experiment.

In form, in feature, in constitution, no two men are alike, and the same is true in regard to their reaction to drugs. It thus happens that however complete our knowledge of any medicament may be, our knowledge of its action on the individual is incomplete, owing to his own idiosyncrasies. The administration of remedies, then, must always partake of the character of a trial and an experiment, and all that we can demand is that the doctor shall try his best, and that his knowledge shall be as complete as possible.

What we wish to insist upon is that throughout the civilised world medicine at the present time is in a condition of quite tremendous scientific activity. The sleep of ages has been broken up, the streams of scientific life are being poured into every artery and vein of the medical organism, and every week new remedies are being offered to the profession which may or may not be of service in the treatment of disease.

As to the composition of the majority of these, there is not the slightest reticence, and there is thus no professional canon against their general use; but as to the real efficacy of many of them there still remains much doubt, and it is a serious question how far the rank and file of the profession should allow themselves to experiment on their patients on the strength of the unsupported recommendations of the inventors of these drugs. There is urgent and immediate need for some form of control over the introduction of new methods and new remedies which the medical profession itself, in every civilised country, ought to provide for. We do not say that the control should be provided at the cost of individuals or even of medical institutions. On the contrary, since it is demanded in the interests of the public safety, the State itself should meet the necessary cost. But though the State should provide for the expenditure, the organisation must be under medical direction, because it is not only control, but information which must be given, and that in so intelligent and sympathetic a way as to provide a just stimulus to regulated experimentation and research in every conceivable direction.

What is required is an authoritative statement as to the action and safety of the various drugs now thrown upon the market, so that medical men, in their endeavours to relieve their patients, may not have to trust so largely as is now the case to the recommendations given by the salesmen of the drugs in question. No man standing in the presence of suffering and disease can be asked to refrain from using remedies whose efficacy is vouched for by well-known authorities unless some central body will undertake what we consider is now an urgent and pressing duty, and will declare which remedy he may safely give and which he had better let alone.

Experiment there will always be, but what form of experimentation is likely to be most fruitful to the furtherance of medicine as a science and an art? Is it that form which works at random, without objective, without definite aim, and with no guidance but the individual whim of the casual and accidental experimenter? Or is it not rather that form which, guided by experience, works on definite lines towards a definite objective, and submits both its processes and its results to the competent criticism of trained and recognised official authority? The subject is both important and immediately urgent; and we earnestly commend its consideration to the minds of all medical statesmen and philosophers.

